# Comparative Analytical Evaluation of Four Centralized Platforms for the Detection of Mycobacterium tuberculosis Complex and Resistance to Rifampicin and Isoniazid

**DOI:** 10.1128/JCM.02168-20

**Published:** 2021-02-18

**Authors:** Margaretha de Vos, Lesley Scott, Anura David, Andre Trollip, Harald Hoffmann, Sophia Georghiou, Sergio Carmona, Morten Ruhwald, Wendy Stevens, Claudia M. Denkinger, Samuel G. Schumacher

**Affiliations:** a Foundation for Innovative New Diagnostics, Geneva, Switzerland; b Department of Molecular Medicine and Haematology, School of Pathology, Faculty of Health Sciences, University of the Witwatersrand, Johannesburg, South Africa; c Institute of Microbiology and Laboratory Medicine, Department IML Red GmbH, WHO-Supranational Reference Laboratory of Tuberculosis, Munich-Gauting, Germany; d SYNLAB Gauting, SYNLAB Human Genetics Munich, Munich-Gauting, Germany; e National Health Laboratory Services, Johannesburg, South Africa; f Division of Tropical Medicine, Center of Infectious Diseases, University Hospital of Heidelberg, Heidelberg, Germany; UNC School of Medicine

**Keywords:** tuberculosis, diagnosis, multidrug resistance, mycobacterium infections, *Mycobacterium tuberculosis*, diagnostics

## Abstract

Failure to rapidly identify drug-resistant tuberculosis (TB) increases the risk of patient mismanagement, the amplification of drug resistance, and ongoing transmission. We generated comparative analytical data for four automated assays for the detection of TB and multidrug-resistant TB (MDR-TB): Abbott RealTi*m*e MTB and MTB RIF/INH (Abbott), Hain Lifescience FluoroType MTBDR (Hain), BD Max MDR-TB (BD), and Roche cobas MTB and MTB-RIF/INH (Roche). We included Xpert MTB/RIF (Xpert) and GenoType MTBDR*plus* as comparators for TB and drug resistance detection, respectively. We assessed analytical sensitivity for the detection of the Mycobacterium tuberculosis complex using inactivated strains (M. tuberculosis H37Rv and M. bovis) spiked into TB-negative sputa and computed the 95% limits of detection (LOD_95_). We assessed the accuracy of rifampicin and isoniazid resistance detection using well-characterized M. tuberculosis strains with high-confidence mutations accounting for >85% of first-line resistance mechanisms globally. For H37Rv and M. bovis, we measured LOD_95_ values of 3,781 and 2,926 (Xpert), 322 and 2,182 (Abbott), 826 and 4,301 (BD), 10,398 and 23,139 (Hain), and 2,416 and 2,136 (Roche) genomes/ml, respectively. Assays targeting multicopy genes or targets (Abbott, BD, and Roche) showed increased analytical sensitivity compared to Xpert. Quantification of the panel by quantitative real-time PCR prevents the determination of absolute values, and results reported here can be interpreted for comparison purposes only. All assays showed accuracy comparable to that of Genotype MTBDR*plus* for the detection of rifampicin and isoniazid resistance. The data from this analytical study suggest that the assays may have clinical performances similar to those of WHO-recommended molecular TB and MDR-TB assays.

## Abstract (precis)

Failure to rapidly identify drug-resistant tuberculosis (TB) increases the risk of patient mismanagement, the amplification of drug resistance, and ongoing transmission. We generated comparative analytical data for four automated assays for the detection of TB and multidrug-resistant TB (MDR-TB): Abbott RealTi*m*e MTB and MTB RIF/INH (Abbott), Hain Lifescience FluoroType MTBDR (Hain), BD Max MDR-TB (BD), and Roche cobas MTB and MTB-RIF/INH (Roche).

## INTRODUCTION

Drug resistance continues to hamper tuberculosis (TB) control efforts despite the availability of rapid diagnostics and standardized treatment regimens for multidrug-resistant TB (MDR-TB) (resistant to rifampicin [RIF] and isoniazid [INH]). In 2017, approximately 558,000 people were diagnosed with rifampicin-resistant TB, of whom 82% had MDR-TB (1). However, only 24% of all new TB cases diagnosed in 2017 were tested for RIF resistance, one of the most important first-line anti-TB drugs (1). Rapid and accurate drug susceptibility testing is crucial to ensure early initiation of appropriate therapy, and failure to do so increases the risk of patient mismanagement, the amplification of drug resistance, and ongoing transmission.

**FIG 1:**
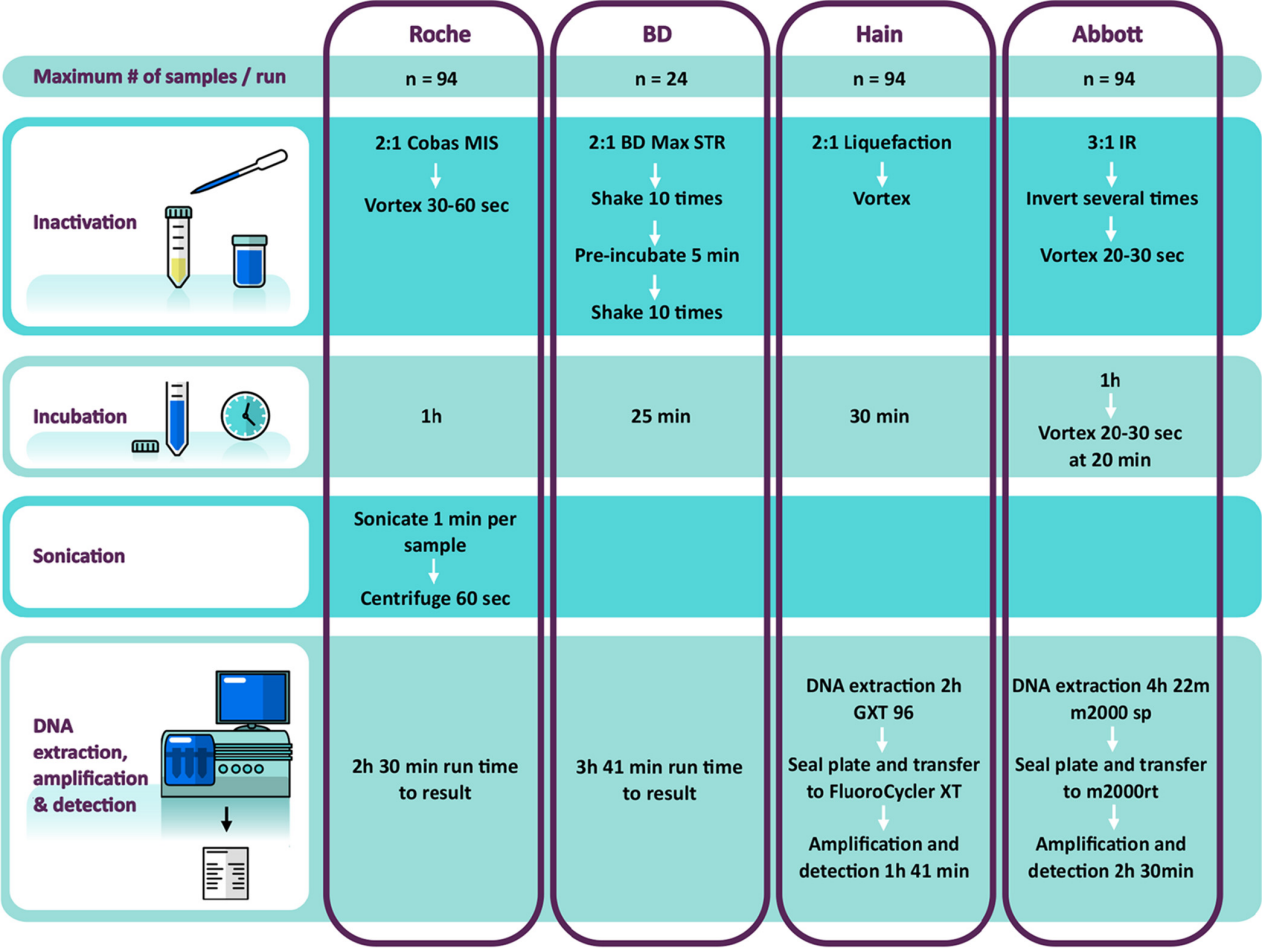
Workflows for each platform for the detection of the M. tuberculosis complex. Each instrument runs the number of samples per run as indicated. Additionally, one positive and one negative control were included in the run, except for the BD Max MDR-TB assay, which does not include external controls. A sample processing control is included in each BD Max MDR-TB run. Run times indicated are for a run with the maximum number of samples. Sonication for the Roche product is done on a separate, dedicated instrument for one sample at a time; centrifugation is done at 3,000 × *g*. Note that the Roche and BD instruments are fully integrated, whereas for the Hain Lifescience and Abbott assays, two separate instruments are used (one for DNA extraction and one for amplification and detection), with a manual transfer step in between. MIS, microbial inactivation solution; IR, inactivation reagent.

The World Health Organization (WHO) has endorsed four molecular assays for the detection of resistance to anti-TB drugs, namely, GenoType MTBDR*plus* (Hain Lifescience, Nehren, Germany) (2), Nipro NTM+MDRTB detection kit 2 (Nipro, Osaka, Japan) (3), the Xpert MTB/RIF assay on the GeneXpert platform (Cepheid, Sunnyvale, CA, USA) (4), and its successor, Xpert MTB/RIF Ultra (5). The GenoType MTBDR*plus* assay is not recommended for use on smear-negative specimens (unless tested from a cultured isolate), and its use is limited by a cumbersome workflow and to laboratories with a high level of infrastructure. Furthermore, the Xpert assays do not include the detection of INH resistance, which compromises their utility in settings with high rates of RIF or INH monoresistance (6, 7). Several novel assays have been developed for the detection of the Mycobacterium tuberculosis complex (MTBC) and drug resistance, including four automated TB assays with intended use in centralized (reference or tertiary) laboratories: the Abbott RealTi*m*e MTB and MTB RIF/INH resistance assays, the Hain Lifescience FluoroType MTBDR assay, the BD Max MDR-TB assay, and the Roche cobas MTB and MTB-RIF/INH assays. These assays target different sites in the MTBC genomes, employ different methodologies for DNA extraction (see Table S1 in the supplemental material), and differ in workflow and throughput (Fig. 1 and Fig. S1). A more detailed description of each of the assays is provided in Appendix S1 in the supplemental material. The assays from Abbott and Roche differ from the other ones where testing is done in a two-step approach and potential resistance markers are determined in a reflex mode only when MTBC is detected in the first run.

Published data from clinical studies to date demonstrate promising diagnostic performances of these assays when used in the appropriate scenarios (8–11). However, most of the available studies have important limitations. For instance, many studies do not include an adequate sample size to achieve precise diagnostic accuracy estimates, and none have tested a large number of resistance mutations in strains of wide geographic variance, as this is typically not feasible in clinical studies (12, 13). In addition, many studies used a sample flow that does not allow for robust comparisons between the index test, the reference test, and WHO-recommended assays as comparators. Generating comparative (direct head-to-head) data against a WHO-recommended assay is an efficient way to establish a portfolio of evidence for new assay solutions (14). Large-scale trials incorporating more than one index assay can be challenging due to specimen volume restrictions. In high-HIV-incidence settings, a significant number of participants may not be able to provide a sputum sample, as TB often does not produce cavities in this population (15, 16). Generating analytical data on the assay limit of detection (LOD) and accuracy in detecting a range of mutations from well-characterized strains can therefore be a useful complementary approach in evaluating new technologies and supporting policy development (12). While all manufacturers include LOD data in the respective assay instructions for use (or package inserts) (Table S2), it is typically not possible to compare LOD estimates from different experiments between assays from different manufacturers due to (i) the lack of universally accepted reference material for MTBC, (ii) variability in LOD studies and experimental design, and (iii) the complexity and variability created by using sputum as a matrix in LOD experiments.

We designed this study to generate comparative data for four centralized assays by (i) assessing the analytical sensitivity expressed as the 95th percentile of the limit of detection (LOD_95_) for the detection of MTBC, using a panel of inactivated strains developed by the Foundation for Innovative New Diagnostics (FIND) and homogenized M. tuberculosis-negative sputum, and (ii) assessing the accuracy of the detection of resistance to INH and RIF using well-characterized MTBC isolates from the FIND biorepository (17). We used Xpert MTB/RIF as a comparator assay in LOD experiments and GenoType MTBDR*plus* as a comparator assay in experiments evaluating the accuracy of resistance detection as these assays have well-characterized clinical performances (18, 19).

## MATERIALS AND METHODS

The external laboratory evaluation on selected strains and panels was carried out at the Department of Molecular Medicine and Haematology’s clinical trial laboratory (Clinical Laboratory Services [CLS]), Johannesburg, South Africa. All centralized assays and instrument handling procedures were performed according to the manufacturers’ instructions and using the manufacturers’ reagents according to standard protocols (Fig. 1; see also Appendix S1 in the supplemental material).

### Assessment of the LOD for MTBC. (i) Materials.

The LOD for MTBC detection was assessed on the four platforms using two inactivated, well-characterized M. tuberculosis strains in defined stock concentrations (5 × 10^7^ genomes/ml). These included a high-IS*6110*-copy-number strain, M. tuberculosis H37Rv, and a low-IS*6110*-copy-number strain, Mycobacterium bovis Z321 (Table S3). These strains were spiked at various concentrations into TB-negative sputum. Pooled and homogenized TB-negative sputum was acquired from adults with pulmonary pathology inconsistent with TB infection presenting to three hospitals in the United States. Although mycobacterial testing of samples was not conducted, samples were collected in nonendemic, low-incidence settings; follow-up was performed with all patients, and a diagnosis other than TB was confirmed. Sputum was provided to the study site in 10-ml aliquots.

### (ii) Methods.

First, we determined a “presumptive LOD” in a series of predefined experiments (detailed descriptions are provided in Appendix S1 and Fig. S1). For the LOD experiment itself, we then selected five concentrations around the presumptive LOD, including two concentrations above the presumptive LOD (with one selected to have 100% positivity) and two concentrations below the presumptive LOD (with one selected to have 0% positivity). Intermediate dilutions (10-fold that of the desired concentration range) were prepared in phosphate buffer and subsequently spiked into the TB-negative sputum aliquots. Each concentration was tested with 20 replicates per assay. One sputum sample mixed with phosphate buffer was used as a negative control. Since an inactivated panel was used for the LOD determination, CFU for each tested dilution could not be determined as the panel was quantified using quantitative real-time PCR against a standard concentration. The results for the LOD experiments therefore represent the numbers of genome copies per milliliter.

### (iii) Statistical analysis.

We used SAS software to estimate the LODs and their 95% confidence intervals (CIs) by using the Poisson binomial probability model as described previously (20). The LOD corresponds to the dilution at a 95% hit rate.

### Assessment of accuracy for the detection of RIF and INH resistance. (i) Materials.

The accuracy for resistance detection was assessed by testing a panel comprising viable M. tuberculosis strains with resistance-conferring mutations in *rpoB*, *katG*, and the *fabG1* (*inhA*) promoter region. MTBC strains characterized by whole-genome sequencing and phenotypic drug susceptibility methods were selected from the FIND biorepository (Table 1) (www.finddx.org) (17). Mutations were selected considering both the confidence (21) in the association between the mutation and phenotypic resistance as well as the frequency of resistance-causing mutations globally (WHO drug resistance surveillance data [personal communication with WHO]).

**TABLE 1:** M. tuberculosis isolate panel used to determine the accuracy of detection of rifampicin and isoniazid resistance

Drug	Gene	Mutation	No. of strains included in the study	Estimated global frequency in resistant isolates (%)^*a*^
Isoniazid	*katG*	S315N	1	3.26
		S315T	16	67.28
	*fabG1* promoter region	−15C/T	5	19.26

Rifampicin	*rpoB*	S450L	5	55.46
		S450W	1	1.66
		S450F	1	0.13
		Q432P	1	0.54
		D435V	1	6.45
		D435G	1	2.09
		D435F	1	0.47
		S441L	1	0.51
		H445D	1	3.97
		H445L	1	1.46
		H445R	2	2.12
		H445Y	1	6.72
		H445G	1	0.13
		L452P	1	3.61
		Q432–433 insertion	1	0.00084

a See reference 21.

### (ii) Methods.

To determine the accuracy of the centralized assays to reproducibly identify RIF and INH resistance, each strain in the M. tuberculosis resistant strain panel (Table 1) was tested in triplicate by all centralized assays. Genotype MTBDR*plus* was used as the comparator test and tested once per strain. The selected strain panel included at least five independent strains for each high-confidence mutation that appears at a frequency of >20% in RIF- and/or INH-resistant strains globally. The panel also included at least one strain for each moderate- and high-confidence resistance mutation with a frequency of less than 20% in RIF- and INH-resistant strains.

### (iii) Statistical analysis.

Two scores were calculated for each assay for RIF and INH. Sensitivity was expressed as a simple proportion (*S_p_*), representing the overall number of mutations detected as a fraction of the total mutations tested in this study. The frequency-weighted sensitivity (*S_w_*) is the number of mutations correctly detected in this study weighted by the estimated frequency with which the specific mutation occurs globally (WHO drug resistance surveillance data [personal communication with WHO]). *S_p_* and *S_w_* scores were also calculated per individual mutation (*S_pi_* and *S_wi_*). Calculations were done using Microsoft Excel.

The scores were defined as follows:

$$S_{p}=100\;\times \;\frac{\sum_{i=1}^{N_{\text{mutation}}}{\text{mutation\_detected}}_{i}}{N_{\text{mutation}}}$$

$$S_{w}=100\;\times \;\frac{\sum_{i=1}^{N_{\text{mutation}}}{\text{frequency\_mutation}}_{i}\times \left(\frac{{\text{number\_strains\_detected}}_{i}}{{\text{number\_strains\_tested}}_{i}}\right)}{\sum^{\;}{\text{frequency\_mutation}}_{i}}$$

where *N*_mutation_ is the total number of mutations tested, *i* is a specific mutation, mutation_detected*_i_* is a logical value set at 1 if mutation *i* is detected and 0 otherwise, frequency_mutation*_i_* is the reported frequency for mutation *i* (reported in Table 4), number_strains_detected*_i_* is the number of strains that tested positive for mutation *i*, and number_strains_tested*_i_* is the number of strains tested for mutation *i*.

## RESULTS

### LOD for MTBC.

Estimates of the LOD per assay and strain at a 95% hit rate are shown in Table 2. The hit rates across concentrations and Poisson binomial curves are provided in Fig. S3 and S4 in the supplemental material. Table S4 shows the LOD per assay and strain relative to Xpert MTB/RIF. For M. tuberculosis H37Rv, the Abbott RealTi*m*e MTB assay showed the lowest LOD at 322 genomes/ml, which was approximately 10 times lower than the LOD of Xpert MTB/RIF (3,781 genomes/ml). The BD Max MDR-TB, Roche cobas MTB, and Hain FluoroType MTBDR assays showed LODs of 826 genomes/ml, 2,416 genomes/ml, and 10,398 genomes/ml, respectively, which were approximately three times higher than the LOD of Xpert MTB/RIF. For M. bovis, the Roche cobas MTB and Abbott RealTi*m*e MTB assays showed lower LOD values of 2,136 genomes/ml and 2,182 genomes/ml than Xpert MTB/RIF (2,926 genomes/ml). The BD Max MDR-TB assay showed an LOD of 4,301 genomes/ml, and the FluoroType MTBDR assay showed the highest LOD, at 23,139 genomes/ml, which was approximately 8 times higher than that of Xpert MTB/RIF.

**TABLE 2:** Limit of detection as determined for each platform for MTBC detection

Mycobacterium	Limit of detection (no. of genomes/ml) at a 95% hit rate (95% CI) for MTBC detection^*a*^				
	Comparator assay (Xpert MTB/RIF)	Centralized assays			
		Abbott RealTi*m*e MTB	BD Max MDR-TB	Roche cobas MTB	Hain Lifescience FluoroType MTBDR
M. tuberculosis H37Rv	3,781^*b*^ (1,974–5,589)	322 (211–432)	826^*b*^^,^^*c*^ (592–1,060)	2,416 (858–3,974)	10,398^*b*^ (6,380–14,416)
M. bovis	2,926^*b*^ (1,991–3,861)	2,182 (1,467–2,897)	4,301^*b*^^,^^*c*^ (3,048–5,555)	2,136^*b*^ (664–3,607)	23,139^*b*^ (15,832–30,446)

a CI, confidence interval.

b No tested dilution had 0% detection.

c No tested dilution had 100% detection.

### Accuracy of RIF and INH resistance detection.

An *S_p_* (simple proportion) score (expressed as the number of strains for which the assays produced valid RIF and INH results divided by the total number of strains tested) was calculated independently for each of the four assays investigated in this study and additionally for GenoType MTBDR*plus* (Table 3). For RIF resistance detection, the *S_p_* scores ranged between 80% (Abbott RealTi*m*e MTB RIF/INH) and 100% (BD Max MDR-TB). Roche cobas MTB-RIF/INH and Hain FluoroType MTBDR had *S_p_* scores of 90% and 97%, respectively. The comparator assay, Genotype MTBDR*plus*, had a score of 95%. For INH resistance, the Abbott RealTi*m*e MTB RIF/INH resistance assay had the highest score of 100%. Roche cobas MTB-RIF/INH and Hain FluoroType MTBDR had *S_p_* scores of 98% and 96%, respectively. BD Max MDR-TB had an *S_p_* score of 95%. The comparator assay, Genotype MTBDR*plus*, had a score of 100%.

**TABLE 3:** Simple proportion and weighted proportion scores per assay calculated for rifampicin and isoniazid^*a*^

Assay	Rifampicin *S_p_* (%)^*b*^ (no. of mutations detected/total no. of mutations tested)	Rifampicin *S_w_* (%)^*c*^	Isoniazid *S_p_* (%)^*b*^ (no. of mutations detected/total no. of mutations tested)	Isoniazid *S_w_* (%)^*c*^
Genotype MTBDR*plus*	95 (19/20)	99	100 (22/22)	100
Abbott RealTi*m*e MTB RIF/INH	80 (48/60)	94	100 (66/66)	100
BD Max MDR-TB	100 (60/60)	100	95 (63/66)	96
Hain FluoroType MTBDR	97 (58/60)	99	96 (63/66)	96
Roche cobas MTB-RIF/INH	90 (54/60)	99	98 (65/66)	NA

a NA, not applicable (could not be calculated as the assay does not discriminate between *inhA* promoter and *katG* mutations).

b Sensitivity was expressed as a simple proportion (*S_p_*), representing the overall number of mutations detected as a fraction of the total number of mutations tested in this study.

c The frequency-weighted sensitivity (*S_w_*) indicates the number of mutations correctly detected in this study weighted by the estimated frequency with which the specific mutation occurs globally (WHO drug resistance surveillance data).

The *S_p_* score calculated for each assay was then weighted by the estimated frequency with which each resistance-conferring mutation is found globally to give a weighted proportion (*S_w_*) score. The *S_w_* was then expressed as a fraction of the total mutation frequency represented by the strain panel (85.3% and 90.2% for RIF and INH resistance, respectively) (Table 1). An *S_w_* score of 94% or higher was achieved by all assays for RIF (Table 3). Genotype MTBDR*plus* had an *S_w_* score of 99% for RIF. For INH resistance, Roche cobas MTB-RIF/INH does not discriminate between *katG* and *inhA* promoter mutations, and therefore, an *S_w_* score could not be calculated. An *S_w_* score of 96% or higher was achieved by all other assays for INH (Table 3). Genotype MTBDR*plus* had an *S_w_* score of 100% for INH.

Table 4 shows the *S_p_* and *S_w_* scores per mutation per assay. The Hain Lifescience Genotype MTBDR*plus* assay failed to detect the one strain with the *rpoB* H445R mutation in one replicate. The Abbott RealTi*m*e MTB RIF/INH resistance assay failed to detect the *rpoB* D435G, S411L, and H445R mutations in all replicates but detected all tested mutations conferring INH resistance. The BD Max MDR-TB assay detected all *rpoB* mutations tested but failed to detect the *katG* S315N mutation in all replicates. The Hain FluoroType MTBDR assay failed to detect one replicate of the *rpoB* S450W mutation and one replicate of the *rpoB* H445R mutation and also failed to detect the *katG* S315N mutation (notably listed as identified in the instructions for use) in all replicates. The Roche cobas MTB-RIF/INH assay failed to detect the D435F mutation and the Q432–433 insertion in all replicates. The Roche cobas MTB-RIF/INH assay does not discriminate between *katG* and *inhA* promoter mutations, and therefore, the individual *S_wi_* score could not be calculated.

**TABLE 4:** Accuracy for the detection of specific mutations conferring resistance to rifampicin and isoniazid^*a*^

Drug	Target	Mutation	Frequency (%)^*b*^	No. of strains	No. of replicates	Abbott RealTi*m*e MTB RIF/INH			BD Max MDR-TB			Hain FluoroType MTBDR			Roche cobas MTB-RIF/INH			Genotype MTBDR*plus*		
						No. detected	*S_pi_* (%)	*S_wi_* (%)	No. detected	*S_pi_* (%)	*S_wi_* (%)	No. detected	*S_pi_* (%)	*S_wi_* (%)	No. detected	*S_pi_* (%)	*S_wi_* (%)	No. detected	*S_pi_* (%)	*S_wi_* (%)
Isoniazid	*katG*	S315T	67.68	16	48	48	100	67.68	48	100	67.68	48	100.0	67.68	NA	NA	NA	16	100	67.68
		S315N	3.26	1	3	3	100	3.26	0	0	0	0	0.0	0	NA	NA	NA	1	100	3.26
	*fabG1*^*c*^	−15C/T	19.26	5	15	15	100	19.26	15	100	19.3	15	100.0	19.26	NA	NA	NA	5	100	19.26

Rifampicin	*rpoB*	S450L	55.46	5	15	15	100	55.46	15	100	55.5	15	100.0	55.46	15	100	55.46	5	100	55.46
		H445Y	6.72	1	3	3	100	6.72	3	100	6.72	3	100.0	6.72	3	100	6.72	1	100	6.72
		D435V	6.45	1	3	3	100	6.45	3	100	6.45	3	100.0	6.45	3	100	6.45	1	100	6.45
		H445D	3.97	1	3	3	100	3.97	3	100	3.97	3	100.0	3.97	3	100	3.97	1	100	3.97
		L452P	3.61	1	3	3	100	3.61	3	100	3.61	3	100.0	3.61	3	100	3.61	1	100	3.61
		H445R	2.12	2	6	0	0	0	6	100	2.12	5	83.3	1.77	6	100	2.12	1	50	1.06
		D435G	2.09	1	3	0	0	0	3	100	2.09	3	100.0	2.09	3	100	2.09	1	100	2.09
		S450W	1.66	1	3	3	100	1.66	3	100	1.66	2	66.7	1.11	3	100	1.66	1	100	1.66
		H445L	1.46	1	3	3	100	1.46	3	100	1.46	3	100.0	1.46	3	100	1.46	1	100	1.46
		Q432P	0.54	1	3	3	100	0.54	3	100	0.54	3	100.0	0.54	3	100	0.54	1	100	0.54
		S441L	0.51	1	3	0	0	0	3	100	0.51	3	100.0	0.51	3	100	0.51	1	100	0.51
		D435F	0.47	1	3	3	100	0.47	3	100	0.47	3	100.0	0.47	0	0	0	1	100	0.47
		S450F	0.13	1	3	3	100	0.13	3	100	0.13	3	100.0	0.13	3	100	0.13	1	100	0.13
		H445G	0.13	1	3	3	100	0.13	3	100	0.13	3	100.0	0.13	3	100	0.13	1	100	0.13
		Q432–433 insertion	<0.01	1	3	3	100	<0.01	3	100	<0.01	3	100.0	<0.01	0	0	0	1	100	<0.01

a *S_pi_*, *S_p_* per individual mutation; *S_wi_*, *S_w_* per individual mutation.

b Frequency of mutation in resistant strain (21).

c *fabG1* promoter region.

## DISCUSSION

This represents the first study to comparatively evaluate the analytical sensitivities of four centralized laboratory assays (the Abbott RealTi*m*e MTB and MTB RIF/INH resistance assays, the Roche cobas MTB and MTB-RIF/INH assays, the Hain FluoroType MTBDR assay, and the BD Max MDR-TB assay) for the detection of MTBC and RIF and INH resistance using WHO-endorsed assays (Xpert MTB/RIF and Genotype MTBDR*plus*) as comparators. Our key findings were that (i) the accuracies of the centralized assays for the detection of resistance to RIF and INH were comparable to that of Genotype MTBDR*plus* and (ii) the analytical sensitivities of all assays were similar to that of Xpert MTB/RIF. These findings were reviewed at a technical expert consultation convened by the WHO and supported the subsequent recommendation for the use of these assays for operational research purposes (22).

Key strengths of this study were the head-to-head comparison of the four centralized assays against well-established comparator assays as well as the use of well-characterized and geographically representative strains. The use of a well-characterized M. tuberculosis resistance panel allowed for direct head-to-head comparisons of the assays for their accuracies in the detection of specific resistance-conferring mutations. We found that all four assays performed equally to MTBDR*plus*, with only low-frequency mutations not being detected by some of the assays. While the total number of strains tested in this study is low, our selection included at least five independent strains for each of the high-confidence mutations that appear at a frequency of more than 20% in RIF- and/or INH-resistant strains globally. In addition, the panel also included at least one strain for each moderate- and high-confidence mutation with a frequency of less than 20%. In total, our selection included mutations that would cover more than 85% of RIF- and INH-resistant strains. These data are highly complementary to data from clinical studies to date despite the fact that the natural occurrence of relevant low-frequency mutations is rare in clinical studies. The data may also guide the uptake and use of these assays in different settings where certain mutations are more or less prevalent.

One strength of this study was the investigation of the analytical sensitivities of the centralized assays using two MTBC strains with different IS*6110* copy numbers. Multicopy insertion elements, such as IS*6110* and IS*1081*, are popular targets for MTBC detection due to their ability to increase the sensitivity of these assays for MTBC detection. Accordingly, the assays targeting IS*6110* (Abbott RealTi*m*e MTB and BD Max MDR-TB assays) showed increased analytical sensitivity compared to Xpert MTB/RIF for M. tuberculosis H37Rv (which contains 15 copies of IS*6110*) while showing results similar to those of Xpert MTB/RIF for M. bovis (which contains a single copy of IS*6110*). The Roche cobas MTB assay showed a slight increase in analytical sensitivity compared to Xpert MTB/RIF for both M. tuberculosis H37Rv and M. bovis. This may be due to the assay targeting five *esx* genes and 16S rRNA for MTBC detection. The Hain FluoroType MTBDR assay showed 8- and 3-times-higher LODs than Xpert MTB/RIF for M. bovis and M. tuberculosis H37Rv, respectively. The higher comparative LODs could be explained by the DNA extraction methodology used by the GXT96 instrument, where intact cells are captured with magnetic beads, washed, and then lysed. The resulting amount of extracellular DNA bound to the magnetic beads is very small. This may also explain the differences observed in the LODs for M. bovis and M. tuberculosis H37Rv if the stocks contained different amounts of free genomic DNA. Both strains contain only one copy of the gene targeted by the FluoroType MTBDR assay, and similar LODs were expected for the two strains. In contrast, the DNA extraction methods used by the other manufacturers (except Xpert MTB/RIF) would capture most extracellular DNA and thus improve analytical sensitivity. This assumption requires further clinical validation, where one may expect a comparable sensitivity for FluoroType MTBDR with improved specificity in patients with a recent history of TB when using the GXT96 instrument for DNA extraction.

Previous analytical studies have noted different LOD values for some of these assays. Analytical studies using culture (counting CFU per milliliter) for quantification will generally show a lower LOD than molecular methods, as any dead bacilli or unbound DNA would not be detected by culture. Chakravorty et al. reported an LOD for Xpert of 112.6 CFU/ml (23), which suggests that the quantification of the panel used in our study may be off by 1 log. The tested panel was, however, designed only for relative comparisons and not to provide exact estimates. Despite this limitation, our findings are in line with studies that showed that Xpert MTB/RIF Ultra had a 10-fold-lower LOD than Xpert MTB/RIF, while the LOD of BD Max MDR-TB was equivalent to that of Xpert MTB/RIF Ultra (23, 24). Analytical sensitivity may, however, vary in different clinical contexts, and as shown by the current study, a degree of uncertainty remains in predicting clinical performance from analytical data. Two studies suggested that Abbott RealTi*m*e MTB may have a clinical performance similar to that of Xpert MTB/RIF (8, 9), while Scott et al. suggested that Abbott RealTi*m*e MTB was more sensitive for MTBC detection in smear-negative, culture-positive specimens than Xpert MTB/RIF (26/35 versus 9/35). In a prospective multicenter diagnostic study conducted by Shah et al., the BD Max MDR-TB assay also showed a performance similar to that of Xpert MTB/RIF (overall sensitivity of 91% versus 90%). In smear-negative specimens, BD Max MDR-TB appeared to have a somewhat higher sensitivity for TB detection than Xpert MTB/RIF (65% versus 59%, with overlapping 95% CIs), although the uncertainty surrounding these estimates was high, with wide 95% confidence intervals (10). A study in South Africa, in a high-HIV-burden setting, also reported that the Roche cobas MTB, Abbott RealTi*m*e MTB, and Xpert MTB/RIF assays (*n* = 294) have similar performances of 94.7% (95% CI, 88% to 98%), 92.6% (95% CI, 85% to 97%), and 91.6% (95% CI, 84% to 96%), respectively. These assays also performed similarly among smear-negative, culture-positive individuals (*n* = 221): 81.8% (95% CI, 60% to 95%), 72.7% (50% to 89%), and 72.7% (50% to 89%), respectively (11).

As expected, the analytical sensitivity of the Abbott and BD assays is highly dependent on the number of IS*6110* copies present in the tested strain. Thus, clinical performance, and comparative performance with Xpert MTB/RIF, which does not target IS*6110*, will also be dependent on the clinical specimens tested and in particular the frequency distribution of IS*6110* elements in the tested samples. We speculate that the smear-negative specimens detected by the Abbott or BD assay that are missed by Xpert may be paucibacillary and/or contain a strain with a high number of IS*6110* copies. Furthermore, since the frequency distribution of IS*6110* copy numbers varies by lineage and the occurrence of different lineages varies across geographical regions, one may expect to find that the sensitivity of these assays (and other assays targeting IS*6110*, such as Xpert MTB/RIF Ultra and Molbio Truenat MTB Plus [Molbio Diagnostics, Goa, India]) varies by country. Further investigation into this area is warranted.

The LOD panel has limitations that need to be taken into consideration when interpreting our results. The panel was initially quantified by real-time PCR (and not by CFU, as the strain stocks were chemically inactivated) and assigned a stock concentration of 5 × 10^7^ genomes/ml. Given that only molecular methods have been used to characterize this panel, the level of extracellular DNA in the stocks was unclear, and this could affect different DNA extraction methods differently. Quantification of M. tuberculosis H37Rv bacteria using Ziehl-Neelsen staining and smear microscopy (performed after the completion of the study) suggested that the stock concentration may be approximately 10-fold lower than expected. If we were to adjust our LOD estimates for this difference, our results largely are consistent with what has previously been reported for Xpert MTB/RIF (23, 25). While there remains uncertainty regarding the efficiencies of the various DNA extraction protocols for M. bovis, all assays have been validated for this strain. Another limitation of the study was that we were not able to include Xpert MTB/RIF Ultra as a comparator, which has an increased sensitivity compared to Xpert MTB/RIF (5). The inclusion of a second comparator would not have been possible due to limited resources (negative sputa and testing panels). We chose to use Xpert MTB/RIF because it is the M. tuberculosis assay with the best-characterized clinical performance (18), the WHO deemed its performance to be sufficient for use as a first-line test and thus may be regarded as a good benchmark, and it is still more frequently used than Xpert MTB/RIF Ultra at the moment. This study also did not assess the performances of the assays to detect silent mutations in *rpoB*, *katG*, and the *inhA* promoter region. Our selection of mutations tested was also limited to the loci targeted by the respective assays, and mutations in other genetic regions would be missed by all assays under investigation. Finally, this study did not assess the LOD for resistance detection. Due to resource constraints, this study also did not assess the interlaboratory reproducibility of the assays for the detection of different resistance mutations.

### Conclusion.

The data from this study suggest that the four centralized assays may have performances similar to that of Xpert MTB/RIF, in line with data from clinical studies. Additional high-quality clinical evaluations are recommended for all assays to strengthen the evidence base. Such studies should include head-to-head comparisons to Xpert MTB/RIF or Xpert MTB/RIF Ultra and should follow guidance on good practice for study design (12, 14). If the high accuracy of these assays is confirmed and a recommendation is issued by the WHO, this would provide high-burden countries with more testing options to improve case detection and resistance testing for TB. As accuracy estimates appear largely similar among the various MTBC and resistance assays, countries will need to determine the best fit for purpose, accounting for TB burden, background epidemiology, HIV coinfection rates, and other operational aspects. Operational aspects such as ease of use, workflow, throughput, and cost, in particular, will be critical to inform decision-making and may also allow for synergies with other disease programs to be realized, increasing overall efficiencies.

## Supplementary Material

Supplemental file 1
