# Supplementary material for: Comparative Analytical Evaluation of Four Centralized Platforms for the Detection of Mycobacterium tuberculosis Complex and Resistance to Rifampicin and Isoniazid
Source: J Clin Microbiol. 2021 Feb 18;59(3):e02168-20. doi: 10.1128/JCM.02168-20 (PMC8106716; doi:10.1128/JCM.02168-20)
Supplement: Supplemental file 1 [file JCM.02168-20-s0001.pdf]

Supplementary appendix

Introduction

Additional information on instruments and assays:

The automated RealTi*m*e MTB assay (Abbott Molecular Inc., DesPlaines, IL, USA) assay can diagnose MTBC in high throughput mode (96 specimens including two assay controls), with software-guided positive specimens reflexed to the RealTi*m*e MTB RIF/INH Resistance assay (24 samples including two assay controls) for full MDR-TB diagnosis within 10.5 hours. DNA extraction and PCR preparation are first performed by the Abbott *m*2000*sp* instrument, after which the PCR plate is sealed and transferred to the *m*2000*rt* instrument for real-time PCR and automated interpretation of the test results. For the diagnosis of MTBC, the assay targets the insertion element IS*6110* as well as the *pab* gene. As a reflex test the detection of resistance to rifampicin and isoniazid the assay targets the *rpoB* gene, and the *katG* gene and *inhA* promoter region, respectively. If the reflex assay is not pre-selected upfront during the MTB test, DNA extraction for the reflex MTB RIF/INH Resistance assay has to be performed separately. Additional information regarding the internal process control and number of assay controls per run can be found in the package insert.

The Hain Lifescience (Hain) FluoroType MTBDR assay (Hain Lifescience, Nehren, Germany) uses Linear After The Exponential (LATE)-PCR amplification and lights-on/lights-off chemistry to detect MTBC by targeting the *rpoB* gene. Detection of resistance to rifampicin and isoniazid is performed through targeting the *rpoB* gene, and the *katG* gene and *inhA* promoter region. The high throughput platform can include up to 96 samples (including assay controls) per run and reports results within 4 hours. The run report includes the specific mutations identified for the three gene targets. DNA extraction and PCR preparation is

performed by the GenoXtract 96 (GXT96) instrument, after which the PCR plate is transferred to the FluoroCycler XT instrument for the FluoroType MTBDR assay.

The Becton Dickson (BD) MAX MDR-TB assay (Becton, Dickinson and Company, BD Life Sciences-Integrated Diagnostic Systems, Québec, Canada) is a real-time PCR assay that can be performed on the BD MAX System (Becton, Dickinson and Company, BD Life Sciences-Integrated Diagnostic Systems, Sparks, MD, USA) to detect MTBC through targeting IS*6110* and IS*1081*. Detection of resistance to rifampicin and isoniazid is performed through targeting the *rpoB* gene, and the *katG* gene and *inhA* promoter region. The assay can include up to 24 sputum samples per run and reports results within 4 hours. The assay also includes a Sample Processing Control that is provided in the Extraction Tube and subjected to extraction, concentration and amplification steps. The Sample Processing Control monitors for the presence of potential inhibitory substances as well as system or reagent failures. Both DNA extraction and the BD MAX MDR-TB assay procedures are done automatically by the BD MAX System. The BD MAX system has the ability to run multiple specimen types and assays in a single run. The BD MAX system has semi-random-access ability as a second run of 24 samples can be loaded once the first batch has been extracted and PCR for the first batch has started.

The Roche cobas MTB assay (Roche Molecular Systems, New Jersey, USA) uses real-time PCR for MTBC detection by targeting 16S rRNA and 5 *esx* genes and can generate results for 96 tests (including assay controls) in one 3.5 hour run. MTBC positive specimens are reflexed to the RIF/INH assay (96 tests including assay controls per run) for MDR-TB diagnosis in an additional 3.5 hours. The assay targets the *rpoB* gene, and the *katG* gene and *inhA* promoter region for detection of resistance to rifampicin and isoniazid. DNA extraction, PCR preparation

and the cobas MTB and MTB-RIF/INH assays are performed in cobas 6800/8800 systems. The cobas 6800/8800 systems allow for random access and the ability to test multiple assays in a run.

Methods

Additional information on workflows:

BD-MAX MDR-TB

The spiked negative sputa and *M. tuberculosis* isolates were treated with the BD MAX-STR reagent (Becton, Dickinson and Company, BD Life Sciences-Integrated Diagnostic Systems, Sparks, MD, USA) to a final ratio of STR:sample of 2:1, mixed by shaking the enclosed sample tube vigorously 10 times, followed by 5 minutes incubation at room temperature. The sample tube was then shaken vigorously 10 times before further incubation for 25 minutes at room temperature. A final volume of 2.5 mL of the STR-treated sample was then transferred to the BD MAX MDR-TB Sample Tube and closed with the septum cap. The BD MAX MDR-TB Unitized Reagent Strip and Sample Tube was subsequently loaded on the BD MAX instrument for analysis.

FluoroType MTBDR

The spiked negative sputa were treated with the Liquefaction reagent to have a final ratio of Liquefaction reagent:sample of 2:1 and mixed by vortexing for 30 to 60 seconds, followed by 15 to 20 minutes incubation at room temperature. A final volume of 700 µl of the liquefied sample was then transferred to a Copan sample and directly used using the GXT96 X2 Extraction Kit on the GenoXtract 96 instrument.

The *M. tuberculosis* isolates were treated with the Inactivation set (Hain Lifescience GmbH, Nehren, Germany) by adding the Inactivation Reagent to the sample to achieve a final concentration of 25% (e.g. 500 µl sample and 167 µl Inactivation Reagent). The treated sample was then mixed by vortexing for 5 to 10 seconds and incubated at room temperature for 30 minutes.

Subsequent DNA extraction and Fluorotype MTBDR PCR preparation by the GenoXtract 96 instrument, the PCR plate was removed from the instrument and sealed. FluoroType MTBDR testing was done in the FluoroCycler XT. The provided positive control was included in each run.

Abbott RealTime MTB and Realtime MTB-RIF/INH

The spiked negative sputa and *M. tuberculosis* isolates were treated with the inactivation reagent (IR) to have a final ratio of IR:sample of 3:1 and mixed by vortexing for 20 to 30 seconds. The mixture was then incubated at room temperature for 1 hour, with mixing of the solution at 20 minutes in the incubation period (vortexing for 20 to 30 seconds). The IR-treated sample was then transferred to a reaction vessel and DNA extraction and PCR preparation was performed using the *m*2000*sp* instrument. RealTi*m*e MTB and Realti*m*e MTB-RIF/INH testing was performed using the *m*2000*rt* instrument. The provided positive and negative controls were included in each run.

Cobas MTB and cobas MTB-RIF/INH

The spiked negative sputa and *M. tuberculosis* isolates were treated with the cobas MIS reagent to have a final ratio of MIS:sample of 2:1 and mixed by vigorous shaking 10 to 20 times or vortexing for 30 to 60 seconds followed by incubation at room temperature for 60 minutes. The treated sample was then transferred to a screw secondary tube and sonicated for 5 minutes, followed by centrifugation for 1 minute at 3000 rcf. The sample tube was then loaded on the cobas 6800 instrument. The provided positive and negative controls were included in each run.

Detailed description of stages of LOD experiments:

(i) Dynamic range: The centralized assay solutions and Xpert MTB/RIF were first evaluated by spiking controlled bacterial concentrations using 10-fold serial dilutions in phosphate buffer. The dilution series included concentrations of 0, 5, 10, 50, 10^2, 10^3, 10^4 and 10^5 genomes/mL. Testing was done in triplicate.

(ii) Target range concentration confirmation: These experiments were done to refine the concentrations to be tested in the LOD experiment. Five concentrations were selected between the lowest dilution showing 100% positivity and the highest dilution showing less than 100% positivity in the dynamic range experiments. Intermediate dilution sets (10x that of the desired concentration range) were first prepared in phosphate buffer and subsequently spiked into the TB-negative sputum aliquots. Each concentration was tested with five replicates. Three replicates of TB-negative sputum mixed with phosphate buffer were used as negative controls.

(iii) In case of inconsistent results observed in the target range concentration confirmation experiments, selection of the target range dilution series and testing was repeated before proceeding to the LOD experiment.

(iv) LOD experiment: From the results of target range concentration confirmation experiments, five concentrations were selected around the estimated LOD. This included two concentrations above (with one selected to have 100% positivity) and two concentrations below (with one selected to have 0% positivity) the estimated LOD. Intermediate dilutions (10x that of the desired concentration range) were prepared in phosphate buffer and subsequently spiked into the TB-negative sputum aliquots. Each concentration was tested with 20 replicates. One sputum sample mixed with phosphate buffer was used as negative control.

Supplementary Tables

Table S1: Mycobacterium genomic regions targeted by the different platforms

Assay	MTBC Target	Multi-copy target	DNA extraction method
Roche cobas MTB	16S rRNA, *esxJ*, *esxK*, *esxM*, *esxP*, *esxW*	No; 6 single copy targets	Bacterial cell lysis is done chemically (lysis reagent), enzymatically (proteinase) and physically (sonication). Released bacterial DNA is captured by magnetic glass particles.
BD MAX MDR-TB	IS*6110*, IS*1081*	Yes	Bacterial cell lysis are done chemically and by heat. Released nucleic acids are then captured by magnetic affinity beads.
Hain FluoroType MTBDR	*rpoB*	No	Capturing of intact cells to magnetic beads, from where the cells are washed and then lysed.
Abbott RealTi*me* MTB	*pab*, IS*6110*	Yes	Cell lysis and capturing of bacterial DNA to magnetic micro particles.

Table S2: Limit of detection and clinical performance as per instructions for use

Assay	LOD as per IFU (CFU/mL) for MTBC detection	Performance as per IFU			
		Overall Sensitivity	Sensitivity Smear-positive	Sensitivity Smear-negative	Overall Specificity
Roche cobas MTB	8.8	92.2-94.9%*	98.9-99.3%*	78.4-86.6%*	97.2-97.9%*
FluoroType MTBDR	14-24*	-	-	-	-
BD MAX MDR-TB	0.5	94.7-96.9%*	98.3-99.2%*	88.5%-90.3%*	94.7%-96.9%*
Abbott RealTi*me* MTB	17	93%	99	81	97%
Xpert MTB/RIF	131	-	99.7%	76.1%	98.8%

- = not reported in IFU; LOD = Limit of Detection; IFU = Instructions for Use; *Depending on sample type (raw and processed sputum)

Table S3: Copy number per MTBC target characteristics per strain used in the limit of detection evaluation

	Copy number per MTBC target	
**Target**	***M. tuberculosis* H37Rv**	***M. bovis***
16S rRNA	1	1
esxJ, esxK, esxM, esxP, esxW	1 copy of each	1 copy of *esxJ*, *esxK*, *esxM*
IS*6110*	15	1
IS*1081*	6	6
rpoB	1	1
pab	1	1

Table S4: Limit of detection relative to Xpert MTB/RIF for MTBC detection

	**Limit of detection for MTBC detection relative to Xpert MTB/RIF**				
	**Comparator assay**	**Centralized assays**			
	**Xpert MTB/RIF**	**Abbott RealTi*me* MTB**	**BD MAX MDR-TB**	**Roche cobas MTB**	**Hain Lifescience FluoroType MTBDR**
***M. tuberculosis* H37Rv**	1	0.09	0.22	0.64	2.75
M. bovis	1	0.75	1.47	0.73	7.91

Table S5: Ziehl-Neelsen staining and smear microscopy results for a dilution series of the H37Rv panel strain used in the analytical evaluation

**H37Rv concentration (genomes/mL)**	**AFB microscopy result**
10^8 genomes/mL	Positive smear 3+
10^6 genomes/mL	Scanty
10^5 genomes/mL	Negative

Supplementary figures

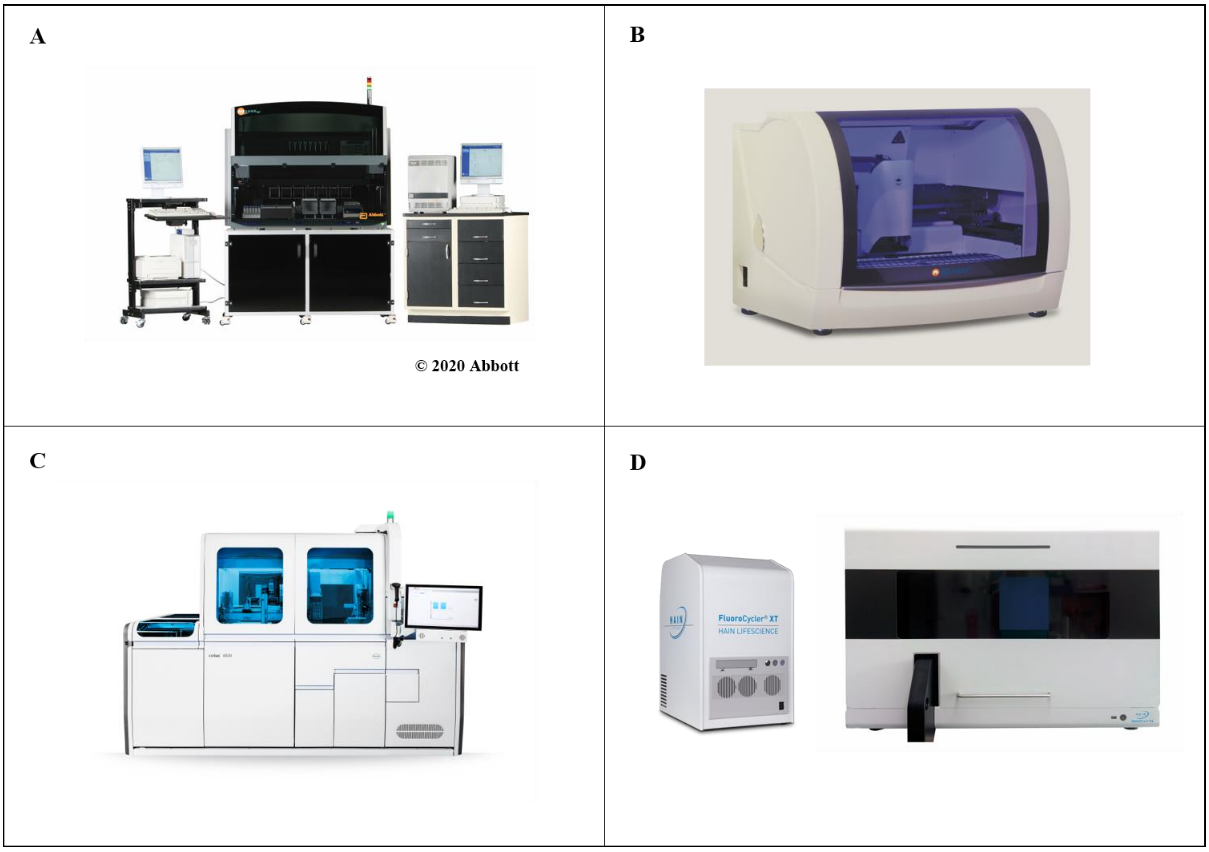

Figure S1: Centralized TB assays for the detection of TB and resistance to rifampicin and isoniazid: (A) Abbott *m*2000*sp* and *m*2000*rt* [Dimensions: *m*2000*sp*: 145cm (Width) X 80cm (Depth) X 177cm (Height) (excluding PC and monitor); *m*2000*rt*: 34cm (Width) X 45cm (Depth) X 49cm (Height) (excluding PC with monitor)]; (B) BD MAX™ System [Dimensions: 114cm(Width) X 170cm (Depth) X 84cm (Height)]; (C) Roche cobas® 6800 (required ultra sonicator not shown) [Dimensions: Cobas 6800: 452cm (Width) X 309cm (Depth) X 216cm (Height); Ultra sonicator (not shown): 74cm (Width) X 45cm (Height) X 34cm (Depth)]; (D) Hain Lifescience FluoroCycler® XT and GenoXtract® 96 [Dimensions: GenoXtract 96: 112.3cm (Width) X 82.5cm (Depth) X 77.4cm (Height) (excluding monitor); FluoroCycler XT: 42cm (Width) X 73cm (Height) X 57cm (Depth) (excluding PC with monitor)]. Material is reproduced with permission from Abbott GmbH; Becton, Dickinson and Company; Hain Lifescience and Roche Molecular Systems Inc.

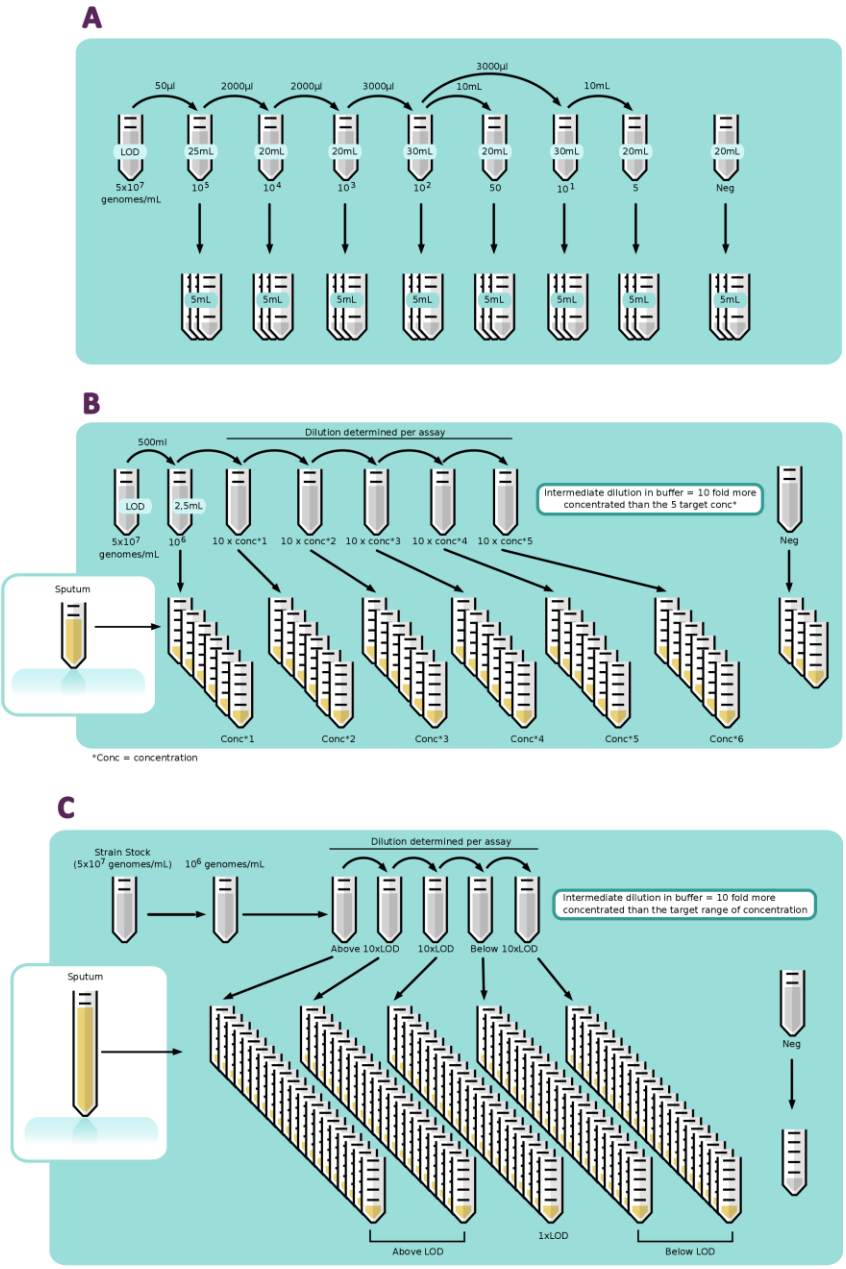

Figure S2: Schematic overview of LOD experiments: (A) Dynamic range experiments: Bacterial stocks were serially diluted in phosphate buffer. Testing was done in triplicate; (B) Target range experiments: Five concentrations were tested with five replicates in sputum. Intermediate 10x dilutions were first made in phosphate buffer and subsequently spiked into sputum; (C) LOD experiments: Five concentrations were selected around the presumptive LOD. The five selected concentrations included two concentrations above the presumptive

LOD (with one selected to have 100% positivity) and two concentrations below the presumptive LOD (with one selected to have 0% positivity). Intermediate 10x dilutions were first prepared in phosphate buffer and subsequently spiked into the TB-negative sputum aliquots.

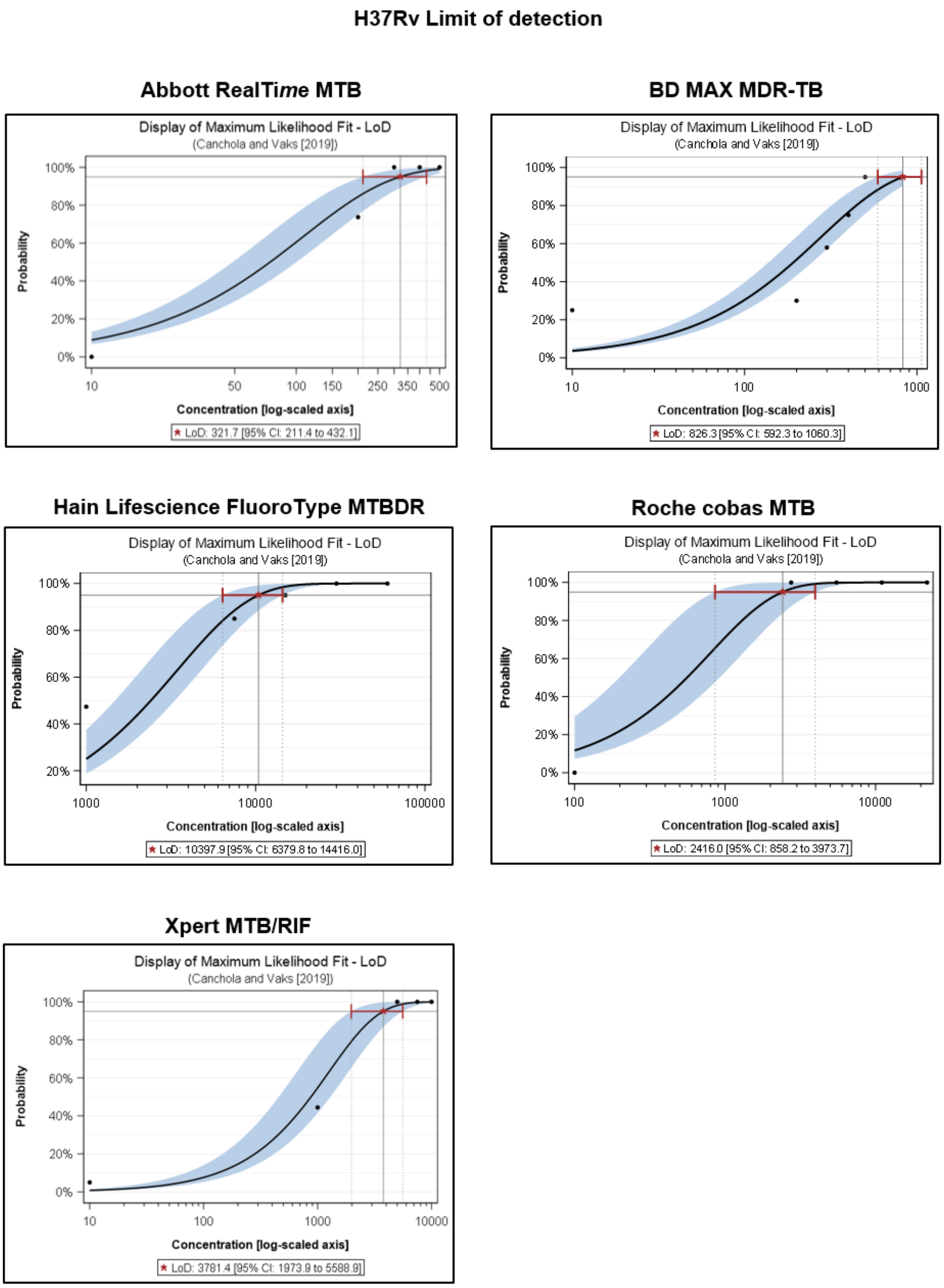

Figure S3: LOD$_{95}$ of Abbott Real*Time* MTB, BD MAX MDR-TB, Roche cobas MTB, Hain Lifescience FluoroType MTBDR and Xpert MTB/RIF when testing *M. tuberculosis* H37Rv. Red dot: calculated LOD$_{95}$, Red line: 95% confidence interval

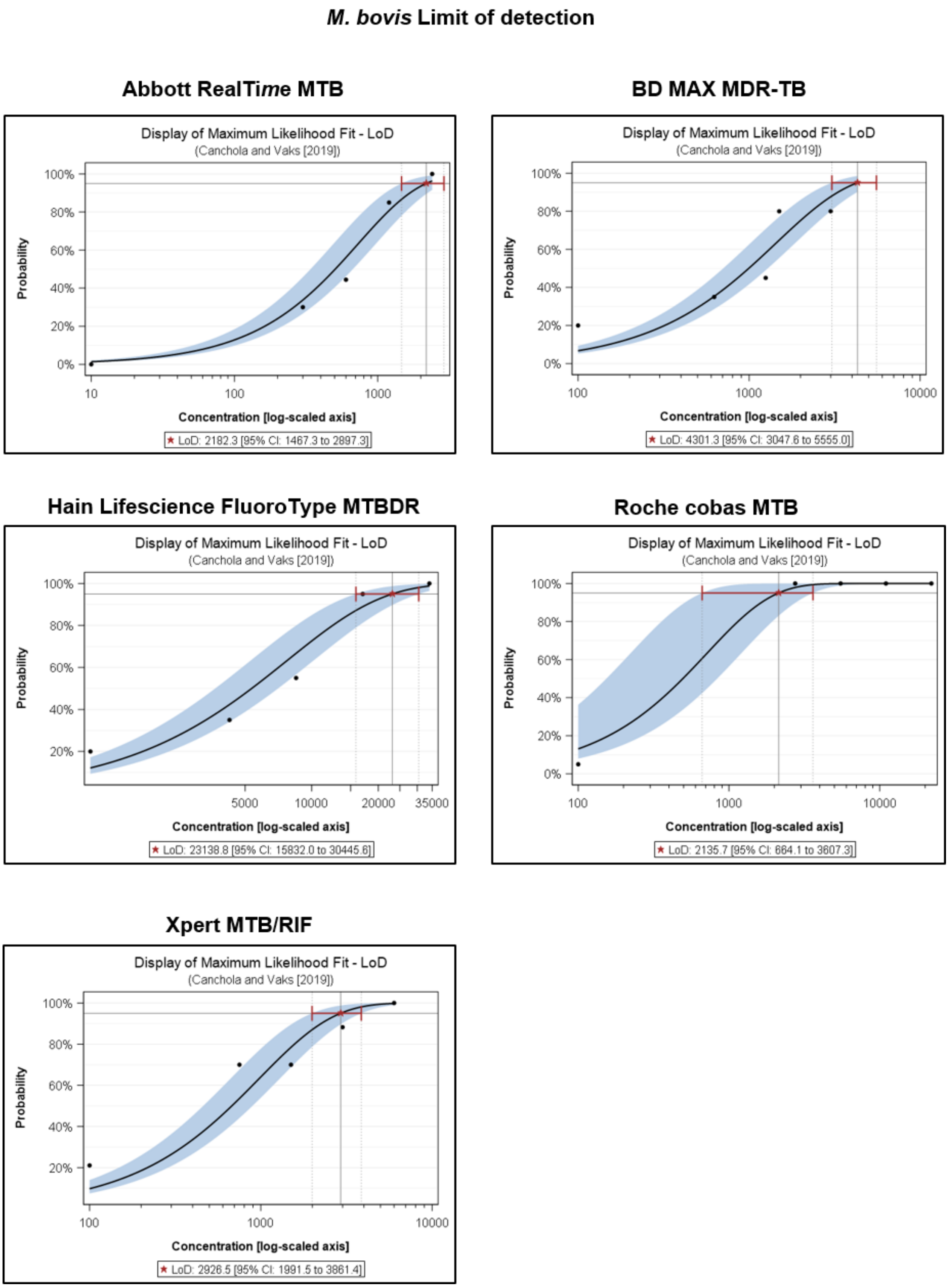

Figure S4: LOD_{95} of Abbott RealTi*me* MTB, BD MAX MDR-TB, Roche cobas MTB, Hain Lifescience FluoroType MTBDR and Xpert MTB/RIF when testing *M. bovis*. Red dot: calculated LOD_{95}, Red line: 95% confidence interval
